# The corruption of power: On the use and abuse of a pre‐trial concept

**DOI:** 10.1113/EP091560

**Published:** 2023-12-05

**Authors:** Ronan M. G. Berg, Robin Christensen, Mathias Ried‐Larsen

**Affiliations:** ^1^ Centre for Physical Activity Research Copenhagen University Hospital – Rigshospitalet Copenhagen Denmark; ^2^ Department of Clinical Physiology and Nuclear Medicine Copenhagen University Hospital – Rigshospitalet Copenhagen Denmark; ^3^ Neurovascular Research Laboratory, Faculty of Life Sciences and Education University of South Wales Pontypridd UK; ^4^ Department of Biomedical Sciences, Faculty of Health and Medical Sciences University of Copenhagen Copenhagen Denmark; ^5^ Section for Biostatistics and Evidence‐Based Research, the Parker Institute Bispebjerg and Frederiksberg Hospital Copenhagen Denmark; ^6^ Research Unit of Rheumatology, Department of Clinical Research University of Southern Denmark, Odense University Hospital Odense Denmark

In various types of study designs used within the field of physiology, so‐called *post hoc* or retrospective power calculations are often provided for data that have already been collected, typically to substantiate claims that the lack of a statistically significant difference for a given outcome likely reflects a type II error, that is, implying that it is due to insufficient sample size. While this old habit remains, it has been widely criticized (Althouse, 2021; Hoenig & Heisey, 2001; Levine & Ensom, 2001). Here, we will briefly highlight why the approach should be avoided while emphasizing that the interpretability in those circumstances can easily be covered by the confidence interval related to the outcome of interest.

In essence, statistical power is exclusively a pre‐trial concept used to inform the design of a study, and – unlike confidence intervals – not a tool for data interpretation. It shows the probability of detecting a statistically significant difference in a given prespecified effect size, given that it really exists within the population from where the study sample was drawn (Cook *et al.*, 2018). Statistical power thus provides critical information about the anticipated sample size needed to foresee a specific probability of   detecting prespecified clinically or biologically relevant differences between different interventions, conditions or groups. The goal is to have a sample size that has sufficiently high statistical power (typically 80% or 90%) for detecting such a difference while setting statistical significance at the level planned for the statistical analysis (usually at the two‐sided 5% level). This should be based on the best available prior knowledge from the background population from which the random sample of the study is drawn, for example, from previous studies on a similar population with similar exposure or, preferably, from pilot data.

Given that the estimates for power calculations are usually based on limited data of varying quality and every so often quite a few assumptions (Schulz & Grimes, 2005), it is not uncommon that they differ from what is observed in the random sample of the study, and it may then be tempting to revisit the statistical power. Obviously, it is often relevant to assess the risk of a type II error in many studies where the *P*‐value of the initial hypothesis test is, for example, above 0.05; despite the ease at which it may be performed in most statistical software packages, a *post hoc* power test is nevertheless not the way forward, as it is both superfluous and misleading. Thus, the statistical analyses of the data provide an effect size, a confidence interval and an exact *P*‐value, within which all relevant information on the power of the study is already represented. As such, a ‘disappointingly high’ *P*‐value will inevitably lead to a low *post hoc* power estimate and vice versa (Hoenig & Heisey, 2001). The pre‐trial statistical power could be perceived as the likelihood that the trial will be successful (i.e., rejecting the null hypothesis as anticipated). It therefore follows that a trial that – for whatever reason – could not reject the null hypothesis was not successful per se. Indeed, a relatively recent study using a comprehensive Monte Carlo‐based simulation clearly demonstrated that when random samples were repeatedly simulated from population distributions, *post hoc* power analyses provided highly variable power estimates that differed markedly from the true statistical power for detecting statistical significance (Zhang *et al.*, 2019).

The evidence‐based medicine movement gained momentum in the early 1990s, transforming medical science by emphasizing meticulous methodologies, particularly the use of accumulated evidence from randomized trials to ensure that clinical practices are founded on the best available evidence (Timmermans & Mauck, 2005). For bridging the ‘translational gap’ between basic and applied sciences, we can learn a lot from evidence‐based medicine when it comes to designing and planning studies, as well as in reporting our findings within the field of physiology. The rigorous design of randomized controlled trials, particularly approaches for minimizing confounding variables to optimize the validity of findings, provides valuable lessons for physiological research. However, it is essential to acknowledge that physiological studies often serve different scientific goals, focusing on understanding basic biological mechanisms, rather than on treatment effects on clinical outcomes, such that alternative methods like the targeted activation or inhibition of biological pathways – e.g., through pharmacological, behavioral, environmental or genetic manipulation – can be more appropriate for controlling confounders. Consequently, if we impose all the standards set forth by evidence‐based medicine on these inherently exploratory investigations, many such studies would become unfeasible due to the constraints of scale, resources and ethical considerations. In this context, it is worth noting that many of the most important discoveries within physiology that define the field to this day deductively focused on individual changes rather than inductive statistical inference, and thus used remarkably small sample sizes. For example, August Krogh and Johannes Lindhard's first recordings of cardiac output and oxygen uptake during maximal aerobic exercise by use of their rebreathing technique were based on measurements on themselves, as well as Marie Krogh, who was doing her own studies in the lab, and a 14‐year‐old child (allegedly the milk boy) (Krogh & Lindhard, 1912). In terms of generalizability, it is commendable that both sexes as well as a ∼35‐year life span were covered in the study, but a sample size (*n*) of 4, would rarely be considered sufficient by today's standards! Likewise, the famous Dallas Bed Rest Study, which reported changes in cardiorespiratory performance from extreme changes in physical activity in five healthy males (Saltin *et al.*, 1968), is often highlighted as one of the most influential studies within exercise physiology. In both of these classic studies, it was clearly not their statistical power that defined their legacy, but rather their careful design and methodology, and the fact that the physiological mechanisms they uncovered have since then been both replicated and reproduced across different study populations and measurement methods. Hence, design is by no means less crucial in physiological research, but a balanced approach is needed, which uses relevant aspects from evidence‐based medicine while also appreciating the unique characteristics and limitations of physiological studies.

We posit that a relatively low statistical power should be accepted as a prerequisite in many physiological studies, provided that the authors are transparent about it. The main risks of underpowered studies are selective analysis and publication bias (Schulz & Grimes, 2005), but this may be hindered by detailed pre‐trial registration that includes the study's working hypotheses, outcome measures and a statistical analysis plan, as well as requirements to report the findings, regardless of how they should turn out. Furthermore, it is important to recognize that even ‘underpowered’ studies may make valuable contributions to the field if they are based on an appropriate study design, methodology that is both internally and externally valid and reliable, and if the data are reported and interpreted appropriately (Schulz & Grimes, 2005). Rather than introducing erroneous idiosyncratic *post hoc* power speculations in this context, we recommend that authors emphasize analysis results by comparing groups with 95% confidence intervals instead of *P*‐values (Christensen *et al.*, 2023). An appropriate interpretation should show awareness that to show ‘no difference’, a smallest clinically or biologically relevant difference must be defined. In physiology, this difference is often obscure, and depends critically on the measurement method (Hartmann *et al.*, 2023). If any biologically or clinically relevant differences are excluded from the width of the 95% confidence interval, a ‘no difference’ or similarity/comparability conclusion is reasonable (Henriksen *et al.*, 2015), such that the risk of a type II error is implied but correctly evaluated as not likely. On the other hand, if a relevant difference is included in the 95% confidence interval (i.e., falls within the two confidence limits), the sample size was probably not large enough (Roos *et al.*, 2018).

In our view, the importance of the tested null hypothesis depends on the smallest biologically or clinically relevant difference, because a conclusion concerning the existence of such a difference is reasonable if the confidence interval excludes all irrelevant differences. By adopting this strategy, we will see less erroneous conclusions that arise from the strictly dichotomous interpretation of the *P*‐value (<0.05) and the lack of an integrated assessment of confidence intervals around the effect size when discussing findings. Just because the individual study does not yield ‘definitive’ evidence, that is, by failing to reject the null‐hypothesis at the 0.05 level, it may still provide valuable additions to the field (Altman, 2012). In the end, it is the collective evidence, and only very rarely the individual study, that provides us with insight.

## AUTHOR CONTRIBUTIONS

Ronan M. G. Berg, Robin Christensen and Mathias Ried‐Larsen conceived the paper and wrote the first draft. All authors made critical revisions, approved the final version of the manuscript, and agree to be accountable for all aspects of the work in ensuring that questions related to the accuracy or integrity of any part of the work are appropriately investigated and resolved. All persons designated as authors qualify for authorship, and all those who qualify for authorship are listed. R.M.G.B. is guarantor of this work and accepts full responsibility for the work and controlled the decision to publish.

## CONFLICT OF INTEREST

The authors declare no conflicts of interest.

## FUNDING INFORMATION

The Centre for Physical Activity Research is supported by TrygFonden Grants ID 101390, ID 20045 and ID 125132. Section for Biostatistics and Evidence‐Based Research, the Parker Institute is supported by a core grant from the Oak Foundation (OCAY‐18‐774‐OFIL).
